# Supplementary material for: Simulation-based benchmarking of isoform quantification in single-cell RNA-seq
Source: Genome Biol. 2018 Nov 7;19:191. doi: 10.1186/s13059-018-1571-5 (PMC6223048; doi:10.1186/s13059-018-1571-5)
Supplement: Supplementary file 1 — Supplementary Figures. This file contains all of the supplementary figures for this paper. (PDF 2136 kb) [file 13059_2018_1571_MOESM1_ESM.pdf]

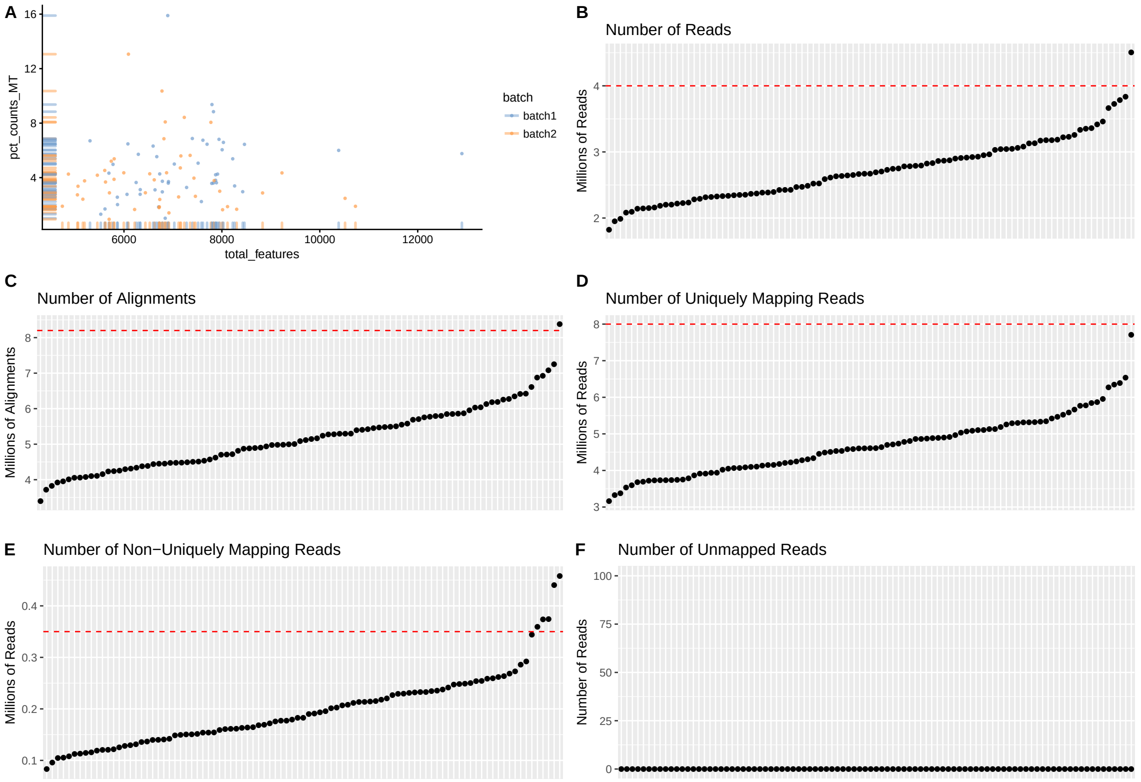

Figure S1: Plots of quality control statistics for the BLUEPRINT B lymphocytes. In all of these plots, one point represents one cell. Based on these plots, cells with more than 10% of reads mapping to mitochondrial RNA, more than 4 million reads, more than 8.2 million alignments, more than 8 million uniquely mapping reads or more than 350,000 non-uniquely mapping reads were removed. Dashed red lines indicate the thresholds selected to remove cells. A: Percentage of reads mapping to mitochondrial RNA. Graph produced using the scater package[1]. B: Number of reads per cell. C: Number of alignments per cell. D: Number of uniquely mapping reads per cell. E: Number of non-uniquely mapping reads per cell. F: Number of unmapped reads.

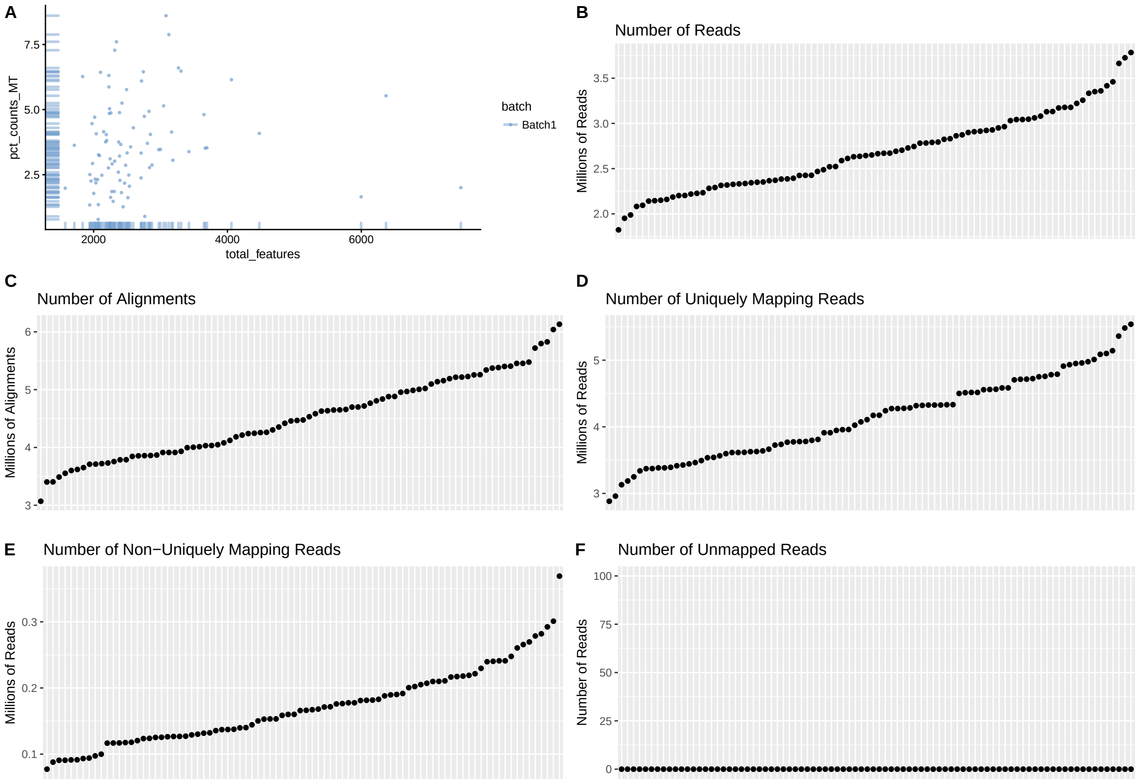

Figure S2: Plots of quality control statistics for the RSEM[2] simulated data based on the BLUEPRINT B lymphocytes. In all of these plots, one point represents one cell. Based on these plots, cells with more than 10% of reads mapping to mitochondrial RNA were removed. Dashed red lines indicate the thresholds selected to remove cells. A: Percentage of reads mapping to mitochondrial RNA. Graph produced using the scater package. B: Number of reads per cell. C: Number of alignments per cell. D: Number of uniquely mapping reads per cell. E: Number of non-uniquely mapping reads per cell. F: Number of unmapped reads.

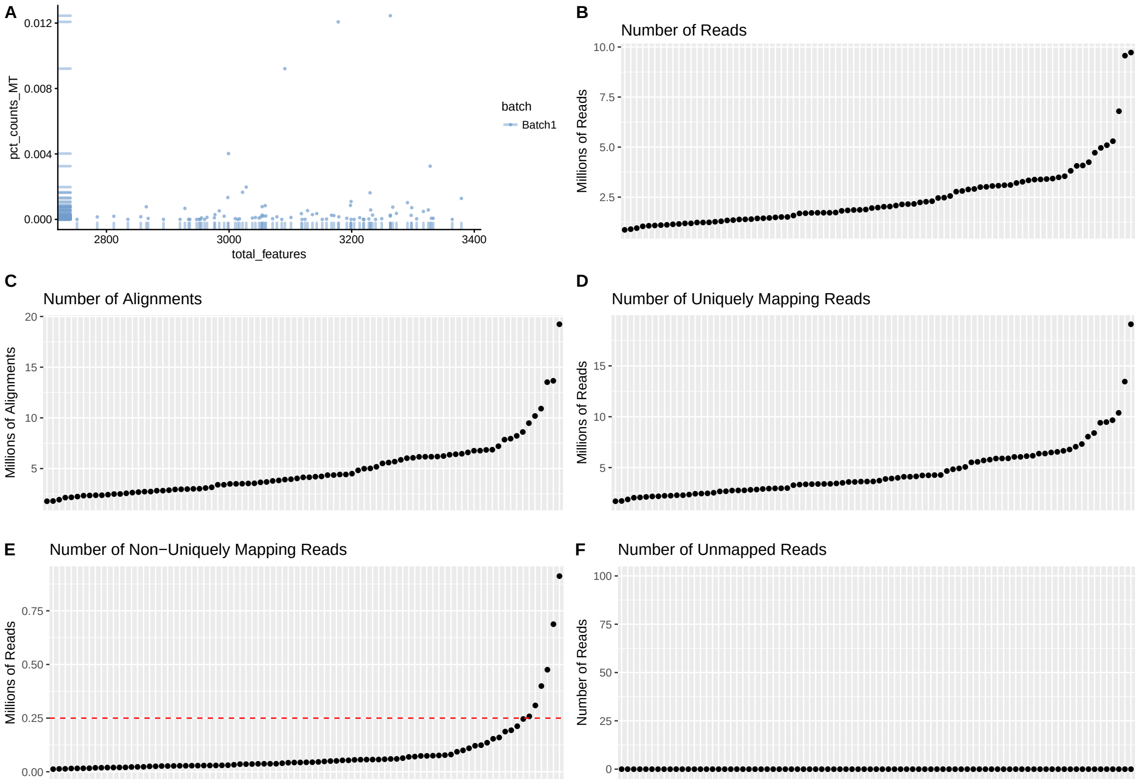

Figure S3: Plots of quality control statistics for the Splatter[3] and Polyester[4] 3' bias simulated data based on the BLUEPRINT B lymphocytes. In all of these plots, one point represents one cell. Based on these plots, cells with more than 250,000 non-uniquely mapping reads were removed. Dashed red lines indicate the thresholds selected to remove cells. A: Percentage of reads mapping to mitochondrial RNA. Graph produced using the scater package. B: Number of reads per cell. C: Number of alignments per cell. D: Number of uniquely mapping reads per cell. E: Number of non-uniquely mapping reads per cell. F: Number of unmapped reads.

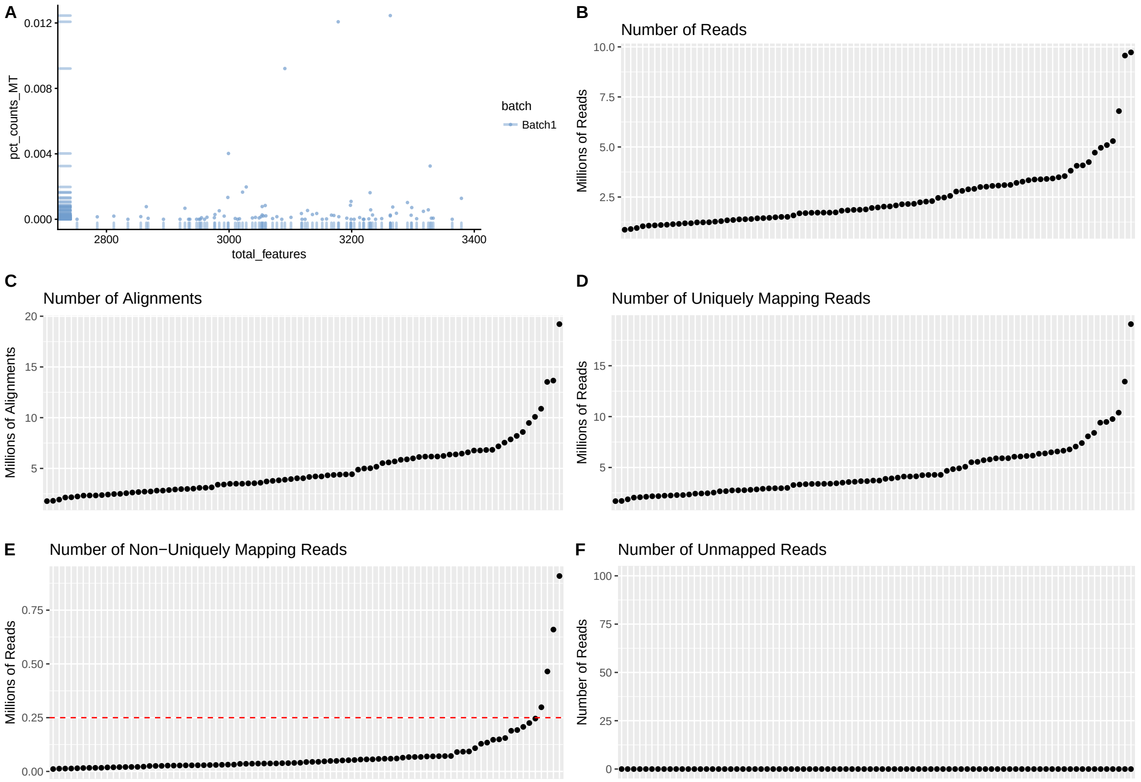

Figure S4: Plots of quality control statistics for the Splatter and Polyester simulated data based on the BLUEPRINT B lymphocytes, simulated with no coverage bias. In all of these plots, one point represents one cell. Based on these plots, no poor quality cells were removed. Based on these plots, cells with more than 250,000 non uniquely mapping reads were removed. Dashed red lines indicate the thresholds selected to remove cells. A: Percentage of reads mapping to mitochondrial RNA. Graph produced using the scater package. B: Number of reads per cell. C: Number of alignments per cell. D: Number of uniquely mapping reads per cell. E: Number of non-uniquely mapping reads per cell. F: Number of unmapped reads.

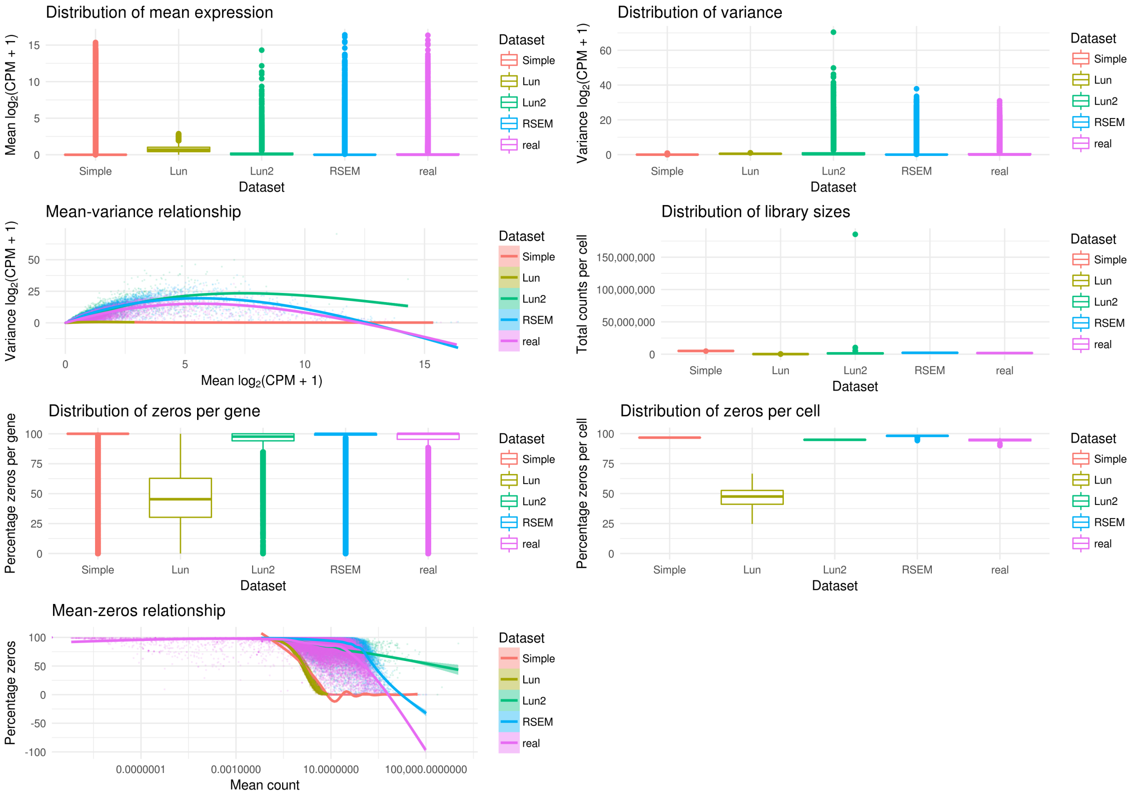

Figure S5: Plots showing characteristics of the different simulation methods included within Splatter, compared with the RSEM simulations and the real data. Based on these plots, the Lun2 method was selected for use in the rest of this paper.

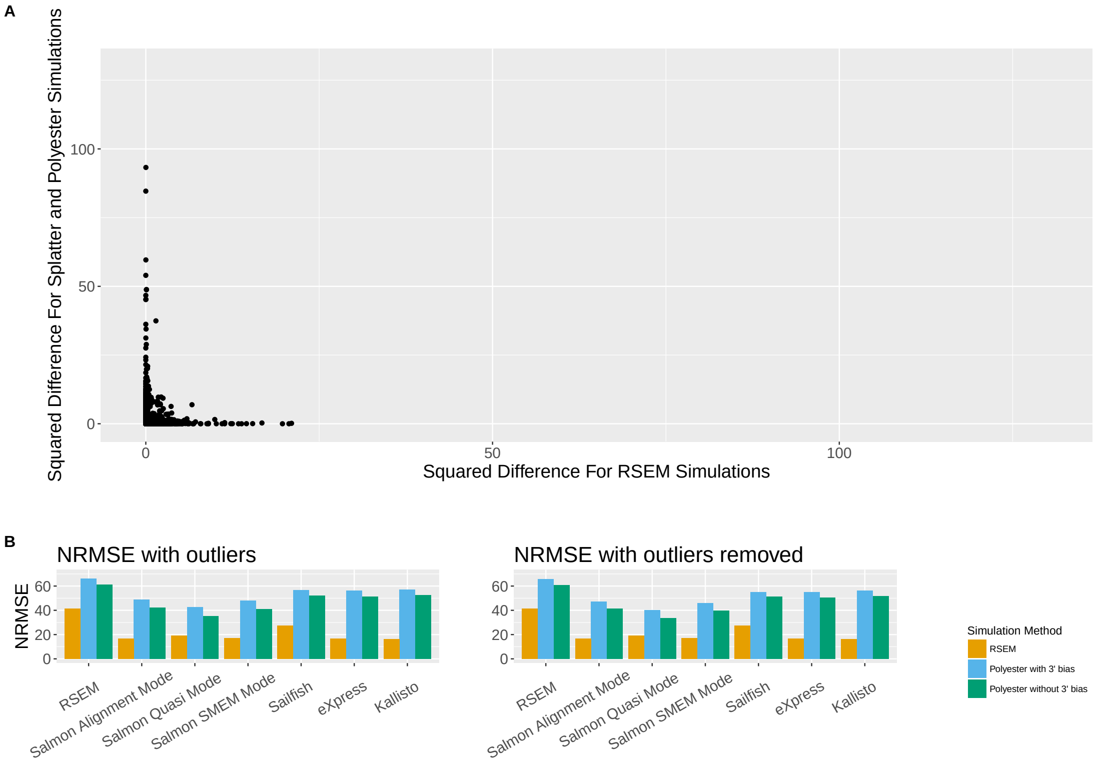

Figure S6: The difference in the NRMSE between RSEM and Splatter and Polyester simulations could not be explained by outliers. A: For each isoform, the mean squared difference between RSEM's expression estimates and the ground truth was calculated for the Splatter and Polyester simulations and for the RSEM simulations. B: The NRMSE when outliers were and were not removed. Based on A, outliers were defined as isoforms with a mean squared difference greater than 30 in the Splatter and Polyester simulations.

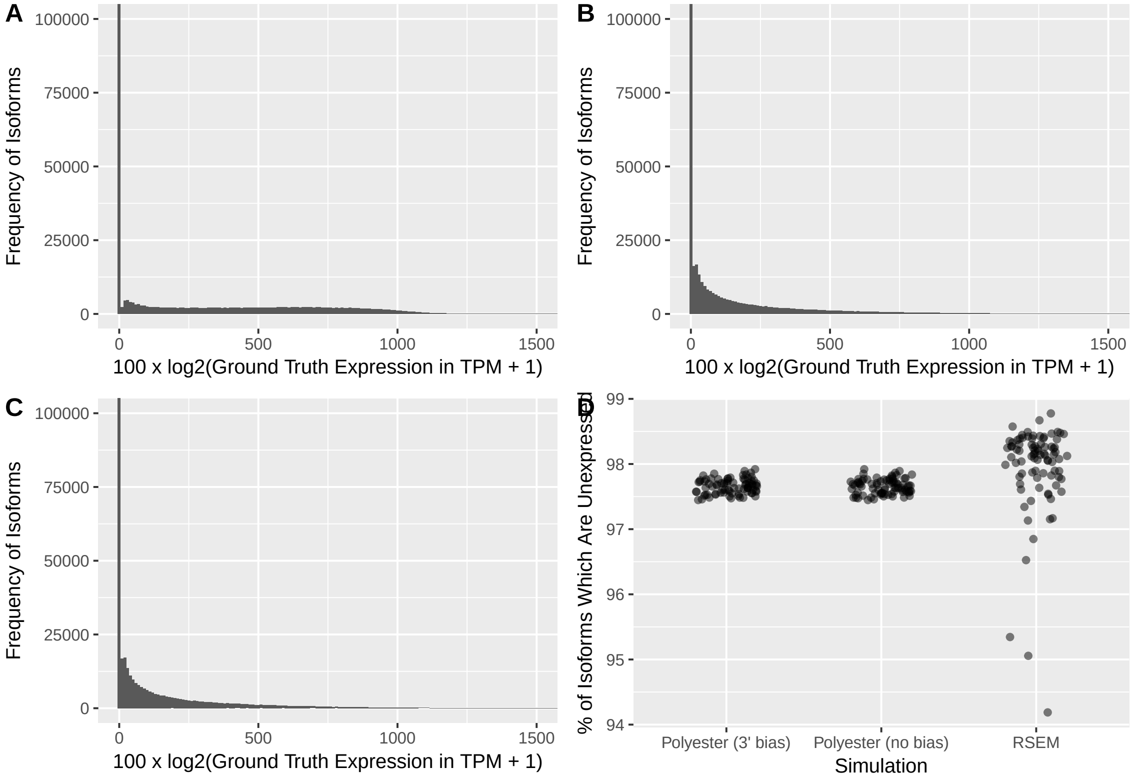

Figure S7: Histograms of ground truth expressions values for different simulation methods. A: Histogram of ground truth expression values for RSEM simulations. B: Histogram of ground truth expression values for Splatter and Polyester simulations with 3' coverage bias. C: Histogram of ground truth expression values for Splatter and Polyester simulations with no coverage bias. D: Percentage of isoforms which are unexpressed (ie. have zero expression) in RSEM and Splatter and Polyester simulations. Each point represents one simulated cell.

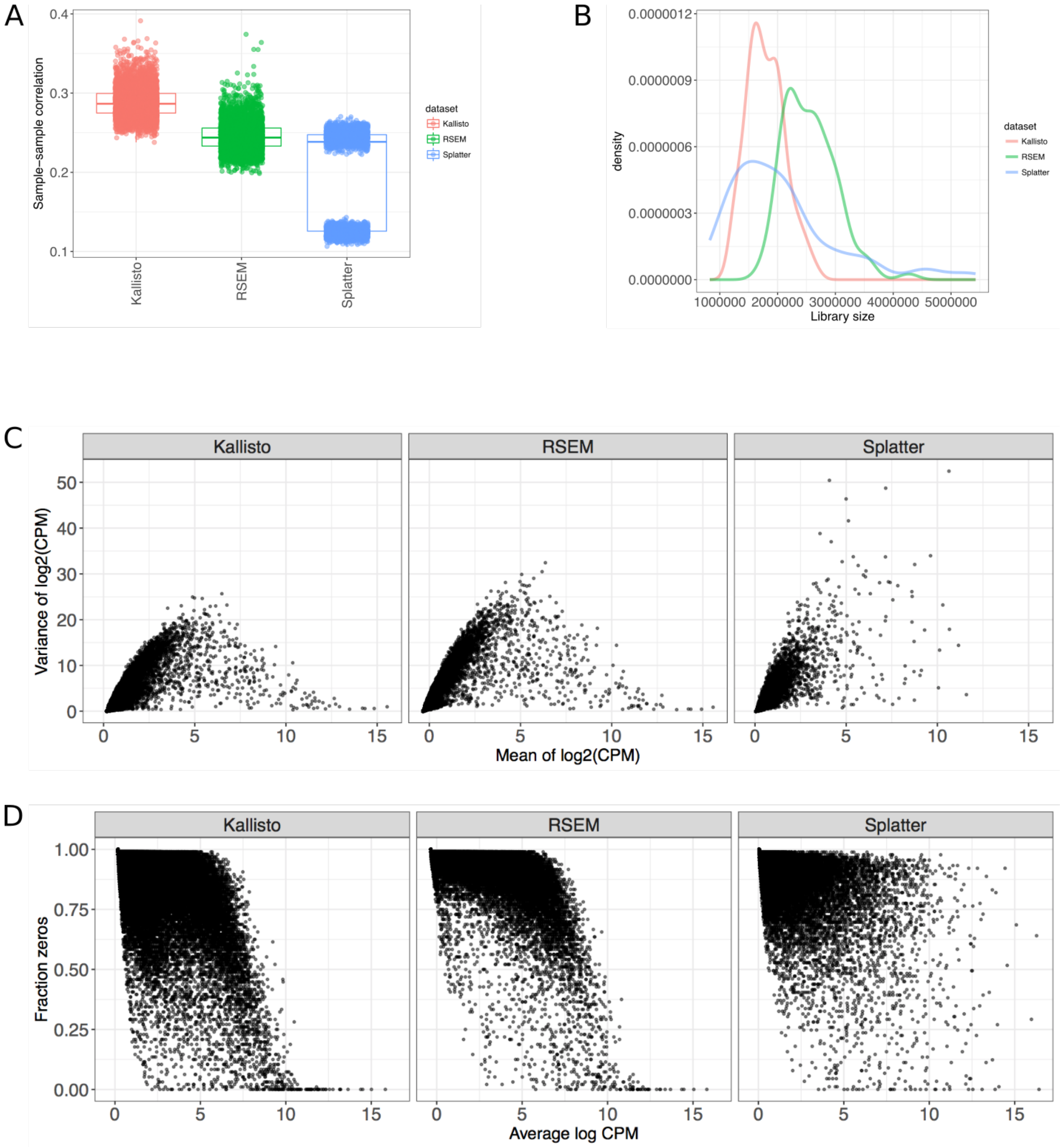

Figure S8: A comparison of the RSEM and Splatter simulations with the real BLUEPRINT data. CountsimQC[5] was used to generate these figures, using expression estimates generated by running Kallisto[6] on the real BLUEPRINT B lymphocytes (red), ground truth expression values from the RSEM simulated data (green) and ground truth expression values from the Splatter and Polyester simulated date (blue). A: Boxplots of sample-sample correlations. Each point represents the Spearman correlation coefficient between two cells. B: Frequency density plot of library sizes. C: Scatter plots of the mean-variance relationship for log2(CPM). D: Scatter plots showing the relationship between the fraction of zeros and the average log CPM.

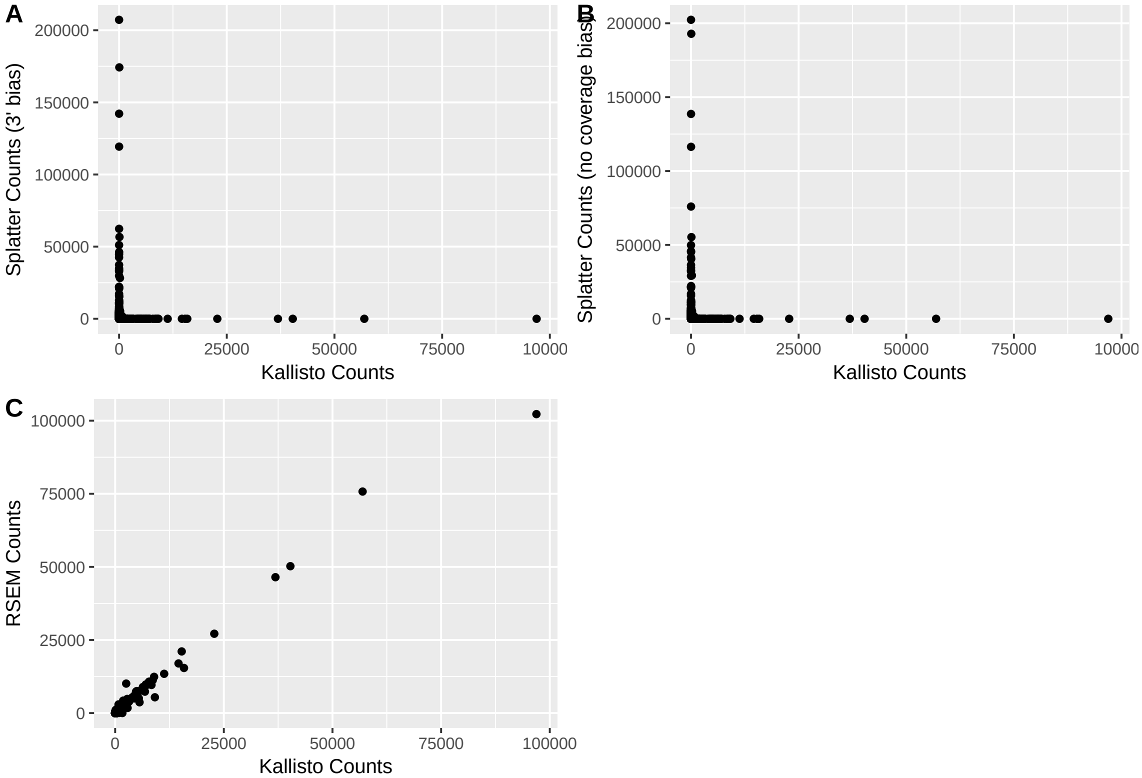

Figure S9: The relationship between expression estimates generated by running Kallisto on the real BLUEPRINT B lymphocytes and the ground truth expression estimates from simulated data. A: Relationship between expression estimates generated by running Kallisto on the real BLUEPRINT B lymphocytes and the ground truth expression values from the Splatter and Polyester 3' bias simulated data. B: Relationship between expression estimates generated by running Kallisto on the real BLUEPRINT B lymphocytes and the ground truth expression values from the Splatter and Polyester simulated data with no coverage bias. C: Relationship between expression estimates generated by running Kallisto on the real BLUEPRINT B lymphocytes and the ground truth expression values from the RSEM simulated data

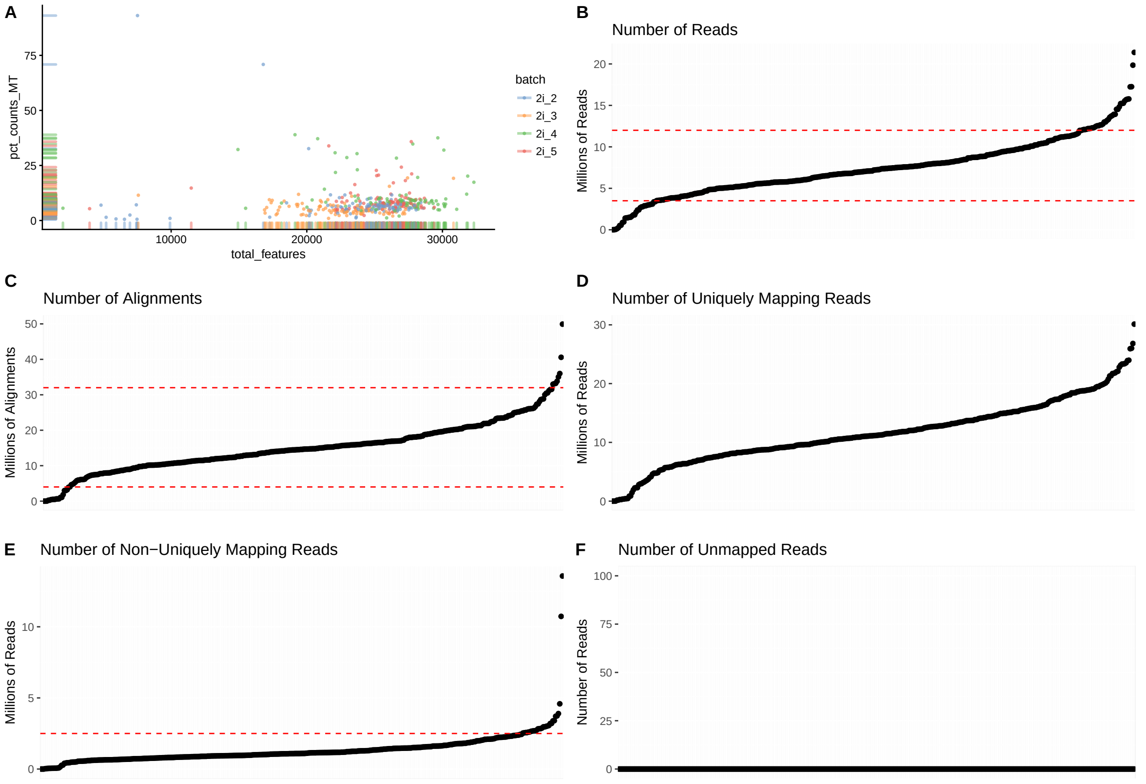

Figure S10: Plots of quality control statistics for mESCs grown in standard 2i media + LIF, published by Kolodziejczyk et al.[7] In all of these plots, one point represents one cell. Based on these plots, cells with more than 10% of reads mapping to mitochondrial RNA, more than 12 million or less than 3.5 million reads, more than 32 million or less than 4 million alignments, or more than 2.5 million non-uniquely mapping reads were removed. Dashed red lines indicate the thresholds selected to remove cells. A: Percentage of reads mapping to mitochondrial RNA. Graph produced using the scater package. B: Number of reads per cell. C: Number of alignments per cell. D: Number of uniquely mapping reads per cell. E: Number of non-uniquely mapping reads per cell. F: Number of unmapped reads.

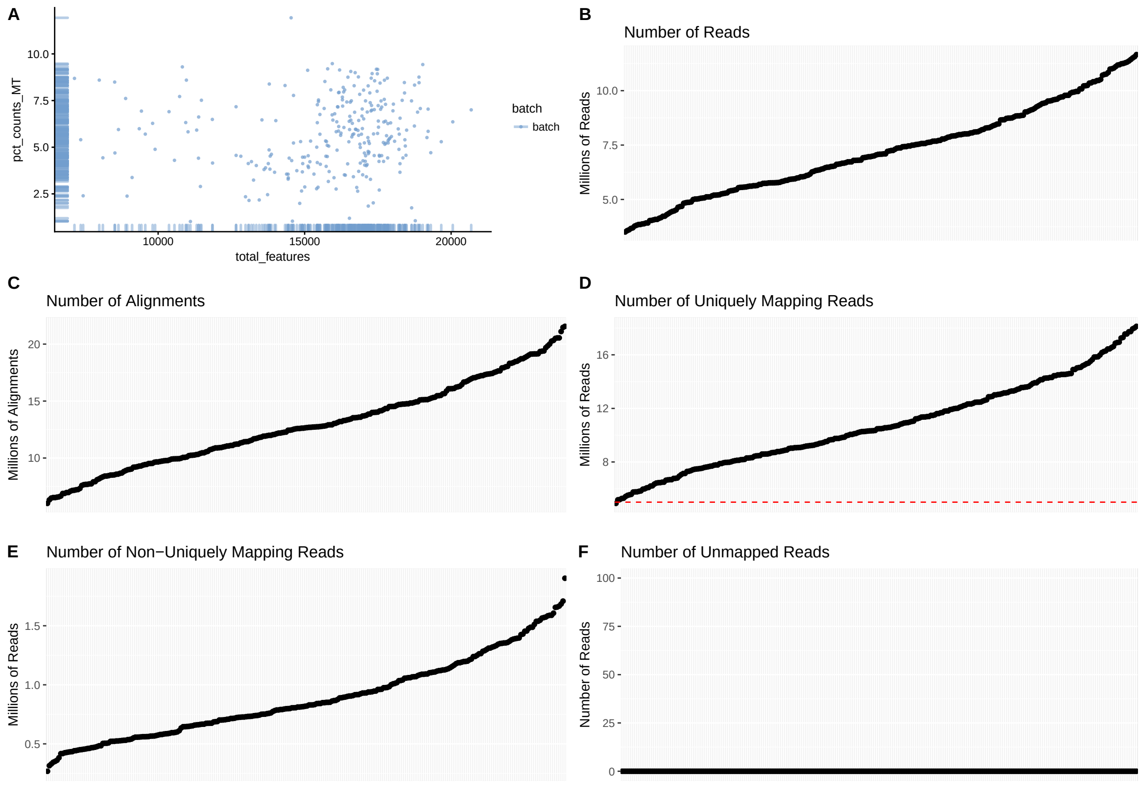

Figure S11: Plots of quality control statistics for simulated mESCs. In all of these plots, one point represents one cell. Based on these plots, cells with more than 10% of reads mapping to mitochondrial RNA or less than 5 million uniquely mapping reads were removed. Dashed red lines indicate the thresholds selected to remove cells. A: Percentage of reads mapping to mitochondrial RNA. Graph produced using the scater package. B: Number of reads per cell. C: Number of alignments per cell. D: Number of uniquely mapping reads per cell. E: Number of non-uniquely mapping reads per cell. F: Number of unmapped reads.

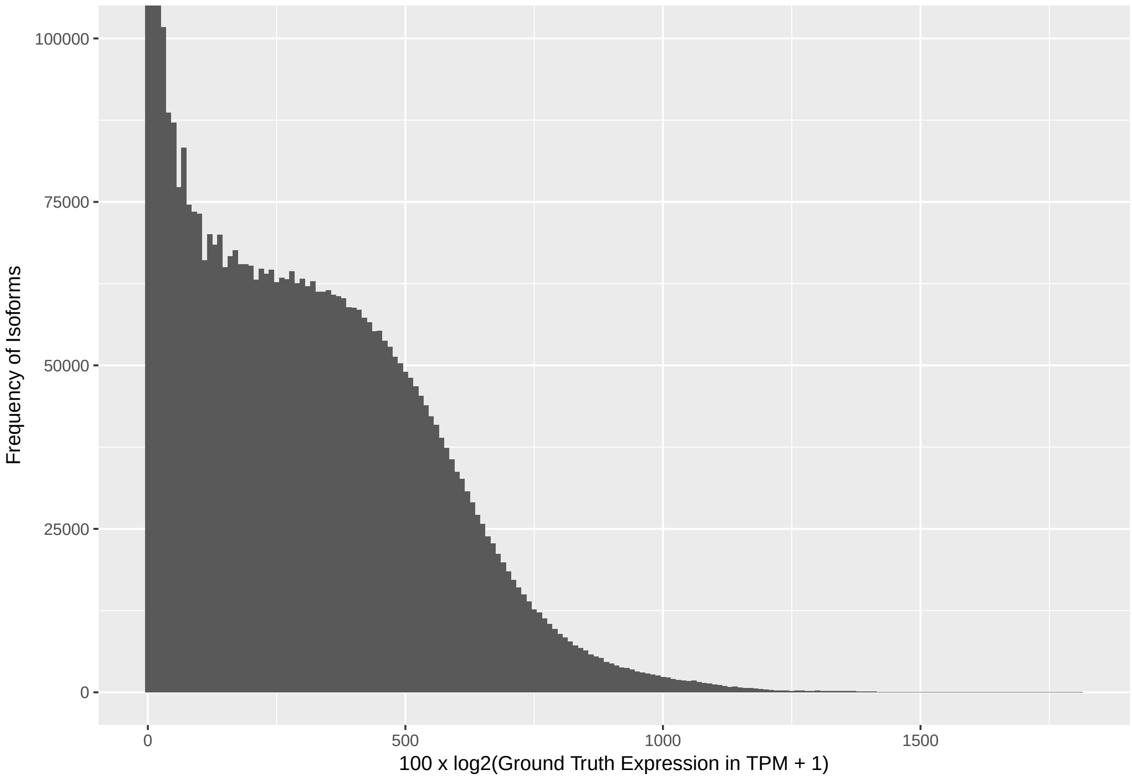

Figure S12: Histogram of ground truth expression values for simulated mESCs.

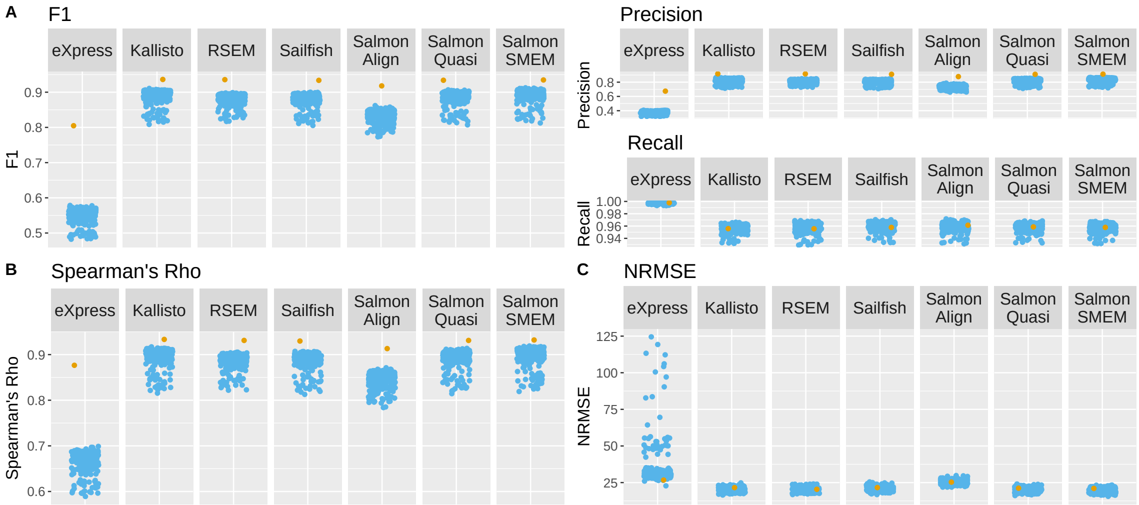

Figure S13: Comparison of the performance of isoform quantification tools on Kolodziejczyk et al. ES cell bulk and single cell RNA-seq data. Each point represents one cell from the scRNA-seq dataset or one bulk RNA-seq experiment. Yellow points represent bulk RNA-seq experiments, blue points represent one cell from the scRNA-seq experiment. (a) F1 score, precision and recall of isoform detection. The F1 score is the harmonic mean of the precision and recall. The precision is the proportion of the isoforms predicted to be expressed by an isoform quantification tool which are expressed. The recall is the proportion of expressed isoforms which are predicted to be expressed by the isoform quantification tool. (b) Spearman's rho. (c) Normalised Root Mean Square Error (NRMSE).

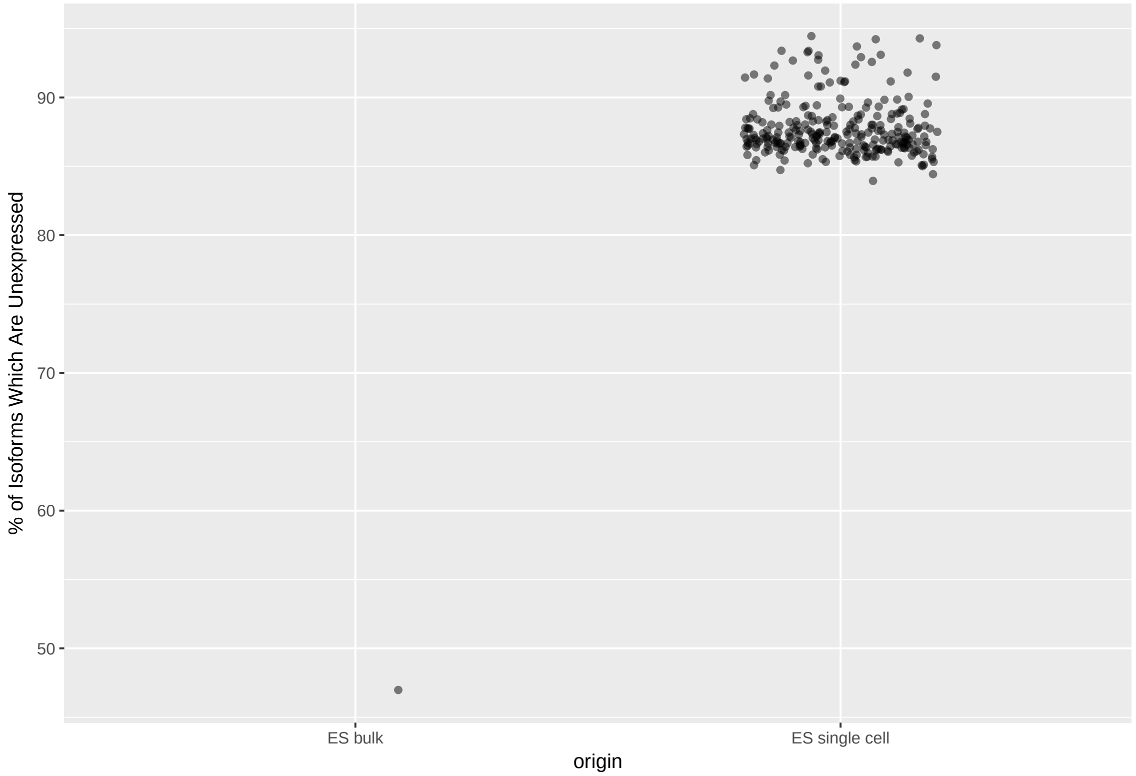

Figure S14: Comparison of the percentage of isoforms which are unexpressed (ie. have zero expression) in BLUEPRINT B lymphocyte and Kolodziejczyk et al. ES cell bulk and scRNA-seq data. For the single cell data, each point represents a simulated single cell. For the bulk data, each point represents a single simulated bulk RNA-seq sample.

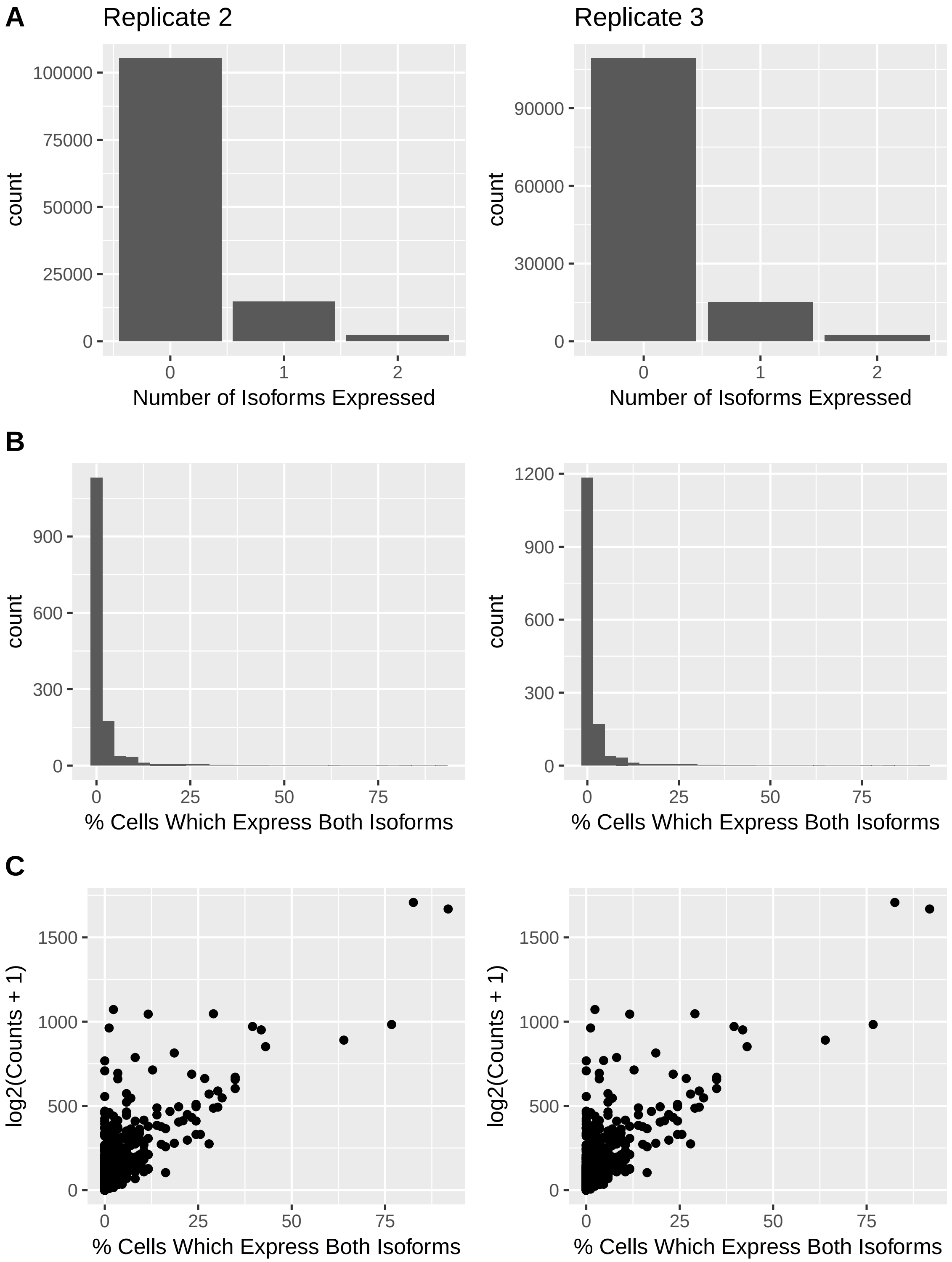

Figure S15: Investigation into how many isoforms are expressed per cell in the scRNA-seq data for genes which express exactly two isoforms using the second (left) and third (right) biological replicates for the BLUEPRINT B lymphocyte bulk RNA-seq data. A: Number of genes which express two isoforms in the bulk RNA-seq data which express zero, one or two isoforms in each cell in the scRNA-seq data. B: Histogram of the percentage of cells which express both the isoforms detected in the bulk RNA-seq data. C: Relationship between the percentage of cells which express both the isoforms detected in the bulk RNA-seq. Spearman's rho is 0.625 for replicate 2 and 0.626 for replicate 3.

References

[1] McCarthy, D.J., Campbell, K.R., Lun, A.T.L., Wills, Q.F.: Scater: pre-processing, quality control, normalization and visualization of single-cell rna-seq data in r. Bioinformatics **33**(8), 1179–1186 (2017). doi:10.1093/bioinformatics/btw777. Accessed 2017-10-18

[2] Li, B., Dewey, C.N.: Rsem: accurate transcript quantification from rna-seq data with or without a reference genome. BMC Bioinformatics **12**, 323 (2011). doi:10.1186/1471-2105-12-323. Accessed 2017-08-21

[3] Zappia, L., Phipson, B., Oshlack, A.: Splatter: simulation of single-cell rna sequencing data. Genome Biol **18**(1), 174 (2017). doi:10.1186/s13059-017-1305-0. Accessed 2017-11-16

[4] Frazee, A.C., Jaffe, A.E., Langmead, B., Leek, J.T.: Polyester: simulating rna-seq datasets with differential transcript expression. Bioinformatics **31**(17), 2778–2784 (2015). doi:10.1093/bioinformatics/btv272. Accessed 2017-08-21

[5] Soneson, C., Robinson, M.D.: Towards unified quality verification of synthetic count data with countsimqc. Bioinformatics (2017). doi:10.1093/bioinformatics/btx631. Accessed 2017-10-09

[6] Bray, N.L., Pimentel, H., Melsted, P., Pachter, L.: Near-optimal probabilistic rna-seq quantification. Nat Biotechnol **34**(5), 525–527 (2016). doi:10.1038/nbt.3519. Accessed 2017-08-21

[7] Kolodziejczyk, A.A., Kim, J.K., Tsang, J.C.H., Ilicic, T., Henriksson, J., Natarajan, K.N., Tuck, A.C., Gao, X., Bhler, M., Liu, P., Marioni, J.C., Teichmann, S.A.: Single cell rna-sequencing of pluripotent states unlocks modular transcriptional variation. Cell Stem Cell **17**(4), 471–485 (2015). doi:10.1016/j.stem.2015.09.011. Accessed 2017-08-21

[8] Shekhar, K., Lapan, S.W., Whitney, I.E., Tran, N.M., Macosko, E.Z., Kowalczyk, M., Adiconis, X., Levin, J.Z., Nemesh, J., Goldman, M., McCarroll, S.A., Cepko, C.L., Regev, A., Sanes, J.R.: Comprehensive classification of retinal bipolar neurons by single-cell transcriptomics. Cell **166**(5), 1308–132330 (2016). doi:10.1016/j.cell.2016.07.054. Accessed 2017-08-21
